# Social responsibility perspective in public response to the COVID-19 pandemic: a grounded theory approach

**DOI:** 10.1186/s12889-022-12819-4

**Published:** 2022-03-09

**Authors:** Lee Lan Low, Seng Fah Tong, Ju Ying Ang, Zalilah Abdullah, Maimunah A Hamid, Mikha Saragi Risman, Yun Teng Wong, Nurul Iman Jamalul-lail, Kalvina Chelladorai, Yui Ping Tan, Yea Lu Tay, Awatef Amer Nordin, Amar-Singh HSS

**Affiliations:** 1 Institute for Health Systems Research, National Institutes of Health Malaysia, Ministry of Health Malaysia, B2, Jalan Setia Murni U13/52 Seksyen U13, Bandar Setia Alam, Selangor 40170 Shah Alam, Malaysia; 2 Department of Family Medicine, Faculty of Medicine, Universiti Kebangsaan Malaysia, Jalan Yaacob Latif, Bandar Tun Razak, 56000 Cheras, Kuala Lumpur, Malaysia; 3 Clinical Research Centre, Hospital Raja Permaisuri Bainun, Ministry of Health Malaysia, Level 4, Ambulatory Care Centre (ACC), Jalan Raja Ashman Shah, 30450 Ipoh, Perak, Malaysia; 4 Department of Community Medicine, School of Medicine, International Medical University Malaysia, No. 126, Jalan Jalil Perkasa 19, Bukit Jalil, 57000 Kuala Lumpur, Malaysia; 5 Galen Centre for Health and Social Policy, Suite C-13A-12, Block C, The Scott Garden SOHO, Jalan Klang Lama, 58100 Kuala Lumpur, Malaysia

**Keywords:** Pandemic, COVID-19, Social responsibility, Societal role, Infection risk, Theoretical model, Qualitative, Malaysia

## Abstract

**Background:**

Combating viral outbreaks extends beyond biomedical and clinical approaches; thus, public health prevention measures are equally important. Public engagement in preventive efforts can be viewed as the social responsibility of individuals in controlling an infectious disease and are subjected to change due to human behaviour. Understanding individuals’ perception of social responsibility is crucial and is not yet explored extensively in the academic literature. We adopted the grounded theory method to develop an explanatory substantive theory to illustrate the process of how individual responded to the outbreak from a social responsibility perspective.

**Methods:**

In-depth interviews were conducted among 23 Malaysians either through telephone or face-to-face depending on the participant’s preference. Both purposive and theoretical sampling were used. Participants were invited to share their understanding, perceptions and activities during the COVID-19 pandemic. They were further probed about their perceptions on complying with the public health interventions imposed by the authorities. The interviews were audio-recorded and transcribed verbatim. Data was analysed via open coding, focus coding and theoretical coding, facilitated by memoing, sketching and modelling.

**Results:**

Study findings showed that, social responsibility is perceived within its role, the perceived societal role responsibility. In a particular context, an individual assumed only one of the many expected social roles with their perceived circle of responsibility. Individuals negotiated their actions from this perspective, after considering the perceived risk during the outbreak. The four types of behaviour depicted in the matrix diagram facilitate the understanding of the abstract concept of negotiation in the human decision-making process, and provide the spectrum of different behaviour in relation to public response to the COVID-19 pandemic.

**Conclusions:**

Our study adopted the grounded theory approach to develop a theoretical model that illustrates how individual response to COVID-19 preventive measures is determined by the negotiation between perceived societal role responsibility and perceived infection risk. This substantive theoretical model is abstract, thus has relevance for adoption within similar context of an outbreak.

## Background

COVID-19 caught the world’s attention when it was declared a pandemic by the World Health Organisation (WHO) [1]. With its rapid spread within and between countries, COVID-19 was classified as a public health emergency of international concern that required a systematic international response [2]. COVID-19 is caused by the Severe Acute Respiratory Syndrome Coronavirus 2 (SARS-CoV-2), which is a novel coronavirus similar to SARS-associated coronavirus (SARS-CoV) and Middle East Respiratory Syndrome Coronavirus (MERS-Cov) that infect the respiratory tract and cause severe respiratory infections. In December 2019, the disease was first reported in Wuhan City, China, in a wholesale food market and infected different groups of people such as stall owners, market employees and regular visitors [3]. Previously, Malaysia experienced a Nipah virus outbreak from September 1998 to May 1999 which resulted in 265 human cases affected and substantial economic loss to the pig-farming industry [4, 5]. Other viral disease outbreaks such as H1N1 and SARS have also affected Malaysia, but none have caused as deep an impact on society as the current COVID-19 pandemic. Similar to other countries, Malaysia has faced challenges in containing the virus and in urging the general public to comply with preventive measures introduced by the government. The first measure that was implemented was the restriction of entry visas from Wuhan City after three Chinese citizens tested positive for COVID-19 in Malaysia on 25th January 2020 [6]. Subsequently, with the rapid rise in local cases, a country level lock-down with a Movement Control Order (MCO) was implemented starting on 18th March 2020, raising the alarm of potential threat to national security [7–9].

An MCO is only effective if there is adherence by the public as the virus spreads via human-to-human transmission. With the increasing burden of cases in the country, mirroring the spread that also occurred internationally, an urgent focus of the government was to prevent the spread and reduce the number of infected cases, thus relieving the strain on health resources. Various preventive measures such as the use of face masks, personal hygiene, restriction of travelling and physical distancing were necessary since vaccines are only just becoming available [10]. The outbreak occurred in an unprecedented manner, causing a burden that was felt by every level of the society. Members of society were urged to assume relevant responsibilities in controlling the spread of this disease, by complying with the MCO. Nevertheless, the action of each individual in adhering to the MCO may vary depending on each person’s perceptions of the disease. Thus, solely relying on community mitigation measures during a pandemic has limitations as it is difficult to change habitual behaviour such as hygiene and social interaction [11]. An evaluation of public knowledge and perception of SARS in Hong Kong showed that enhanced personal hygiene and health protective measures relied critically on public psychological responses and widespread perceptions and beliefs of the community at large [12]. The research group found considerable misinformation and false beliefs among the community in Hong Kong at an advanced stage of the SARS epidemic, despite the wide coverage and substantial mass media and public service announcements. Hence, public perception is very important in improving health risk communication, building public trust and cooperating with the government’s preventive effort to control an outbreak [13].

Combating viral outbreaks extends beyond biomedical and clinical approaches; thus, public health prevention measures are equally important. Hence, it is essential to understand the Malaysian public response to COVID-19 preventive measures. Malaysians should practise social responsibility by complying with the Standard Operating Procedure (SOP) and guidelines imposed by the government [9, 14]. Public engagement in preventive efforts can be viewed as the social responsibility of individuals in controlling an infectious disease [15] and are subject to change due to human behaviour. While human behaviour is complex, many health behaviour models have been developed to understand health, behaviour and health system phenomena by incorporating various features of individuals, communities and organisations. Other health behaviour models include the health belief model [16] and the COM-B model [17]. Although the health belief model was established to study health-related phenomena [18–20], it has a limitation that it does not directly incorporate societal features such as social norms and community assumptions, which are assumed in underlying demographic factors [21]. The COM-B model introduced Capability, Opportunity and Motivation into a model by focusing on promoting individual behaviour adherence to public health interventions [22].

Studying human behaviour extends beyond the individual’s level and it involves examining collective efforts and societal participation. Understanding how people conceptualise social responsibility during an outbreak can provide useful information for improving future preventive and control interventions. Hence, this study aims to explore public conceptualisation of social responsibility and to develop a model explaining the role of social responsibility during the early COVID-19 pandemic in Malaysia.

## Method

### Study design

In-line with the aim of developing an explanatory substantive theory explaining the role of social responsibility during the early COVID-19 outbreak, the grounded theory methodology was adopted [23]. An in-depth interview (IDI) was used to capture emic perspectives and provided a detailed account of a person’s view, reactions (personal responsibility) and social processes (social responsibility) among the general public during the early COVID-19 outbreak in Malaysia. The purposive sampling strategy was used with the intention of exploring a range of individuals from various social backgrounds and different experiences encountered in relation to the current pandemic. Subsequently, the theoretical sampling was applied to aid the saturation of the model, which enabled enrichment of data that could illustrate the process of how individuals perceived societal role responsibility within the outbreak environment. This led to the identification of a ‘core category’ for this study and the theoretical saturation.

### Recruitment & participants

**Fig. 1:**
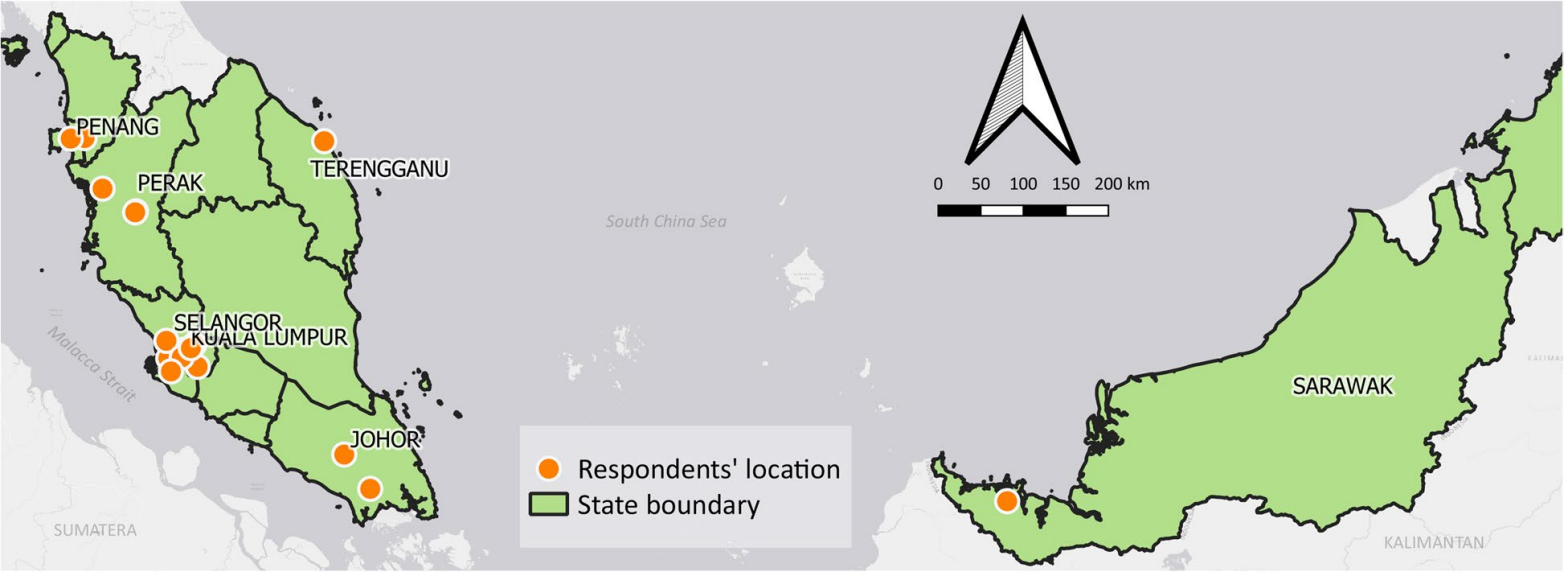
Malaysia map with locations of the selected participants. *Notes: respondents = study participants

Participants were purposively recruited from different demographic backgrounds to elicit their diverse experience during the early COVID-19 outbreak. Potential participants were identified from the social networks of the research team as well as referrals by the social network among their friends. All participants selected resided in different locations within Malaysia when the interview was conducted. In total, 23 Malaysian adults with various sociodemographic were interviewed. Figure 1 shows the locations of study participants.

### Data collection

The interviews were conducted between March to July 2020 by researchers who were trained in qualitative method. The movement restriction order that was implemented during the worsening stages of COVID-19 outbreak in Malaysia [8] limited physical accessibility to conduct face-to-face interviews. This limitation was surmounted by conducting telephone interview. Hence, only one IDI was conducted face-to-face at the workplace based on the participant’s request, while the other 22 IDIs were through telephone interviews. A semi-structured interview guide was developed to facilitate IDI sessions, containing a list of questions pertaining to the understanding of COVID-19, “perceptions on the outbreak progression and consequences of the outbreak on everyday lives and activities”, “experiences and perceptions upon searching for and receiving COVID-19 information”, further probing to explore their “ways in handling the information received and reasons behind their actions”. Participants were also asked about their “activities and perceptions in complying with the public health interventions imposed by authorities during the outbreak”. The interviews sessions lasted between 25 and 110 min. They were conducted either in English, Malay or Mandarin language depending on the participant’s preference. Participant information sheet which included study objective, the purpose of the interview and the interview process was shared through email or WhatsApp message prior to the interview and informed consent was obtained from all participants. Assurance of confidentiality was given that data would be used only for this study. All IDIs were transcribed verbatim and cross-checked against audio recording by another team member to ensure accuracy of the transcripts. Data collection continued until data saturation. Upon interviewing 23 participants, no new emerging theme was observed from two consecutive IDIs. No repeat interviews were carried out since the information gaps were probed in the subsequent interviews.

### Analysis

The interviews were audio-taped as permitted by the participants, transcribed and imported into the Nvivo 11 (Qualitative data analysis computer software) to facilitate data analysis. Participants’ identifiers were replaced with researcher-assigned codes to maintain the anonymity of the transcripts prior to analysis. Data analysis began immediately after the first interview. Theoretical saturation was observed and achieved after 23 interviews, whereby no new categories were found to add further understanding of the complex human behaviour and social responsibility in facing the early COVID-19 outbreak. Data analysis involved various stages of coding, memoing, sketching and modelling. Coding began with open coding, followed by identifying categories and grouping. The coding process started with immersion in the data, whereby the texts were read several times before they were coded. Focus coding was carried out subsequently, followed by a theoretical coding. Eventually, the core category was identified, and the study theoretical framework was conceptualised. The core category “negotiation” was identified based on its ability to subsume the main categories in explaining the entire process of an individual’s conceptualisation of social responsibility and how they exercised their social responsibility.

### Study team

The research team consisted of multi-disciplinary researchers with various experiences, composing those with experience in behavioural sciences (medical anthropologist), public health and clinical research, health systems research and primary care services. Trained in qualitative research and interviewing techniques, all research team members are fluent in English and Malay languages and a few are also fluent in Mandarin.

### Ethical considerations

This study was registered with the National Medical Research Register (NMRR-20-574-54389). Ethical approval was granted by the Medical Research and Ethics Committee, Ministry of Health Malaysia (KKM/NIHSEC/ P20-701(7)). Participants’ identity and data confidentiality were assured throughout the data collection. Informed consent to participate and permission for audio recording were obtained from all participants prior to interviews.

## Results

The results of this study were obtained from in-depth interviews with 23 individuals from the general public between March to July 2020 in Malaysia. Table 1 summarises the characteristics of the participants. More than half were female (69.6%), Malay ethnic group (52.2%) and 78.3% of them had at least a tertiary education level. They ranged from 29 to 73 years old, work as public civil servants (39.1%), in private sector (39.1%) and 21.8% were retirees.

**Table 1:** Demographic characteristics of study participants

Demographics	*N* = 23 (%)
Mean age in years, (range)	46.9 (29–73)
Gender, n (%)	
Male	7 (30.4)
Female	16 (69.6)
Ethnicity, n (%)	
Malay	12 (52.2)
Chinese	6 (26.1)
Indian	4 (17.4)
Siamese	1 (4.3)
Education Level, n (%)	
Primary	1 (4.3)
Secondary	4 (17.4)
Tertiary	18 (78.3)
Occupation, n (%)	
Civil servant	9 (39.1)
Private sector	9 (39.1)
Retirees	5 (21.8)

From the perspective of an individual’s obligation to act for the benefit of society, the participants conceptualised social responsibility from two dimensions: (1) the individual perceived roles in their society, and (2) the individual perceived circle of responsibility. To finally exercise social responsibility would involve individuals considering the perceived risk of infection.

The individual perceived role defines their actions. The extent of the action regarding the level of societal involvement depends on how wide a person casts his or her circle of responsibility in the society. Individuals often hold multiple roles; thus, their respective circles of responsibility vary, as are their actions. The individuals’ perception of infection risk is constructed from their response to information received and their self-efficacy in possible risk modification. Under a pandemic circumstance, the recommended preventive measures such as movement restriction, personal hygiene and the use of protective equipment are negotiated from this perspective of societal role responsibility. The negotiation involves reaching an agreement [24] and in the context whether one would decide to take the preventive measures deemed necessary. This negotiation is produced by the intersection between societal role responsibility and perceived infection risk.

**Fig. 2:**
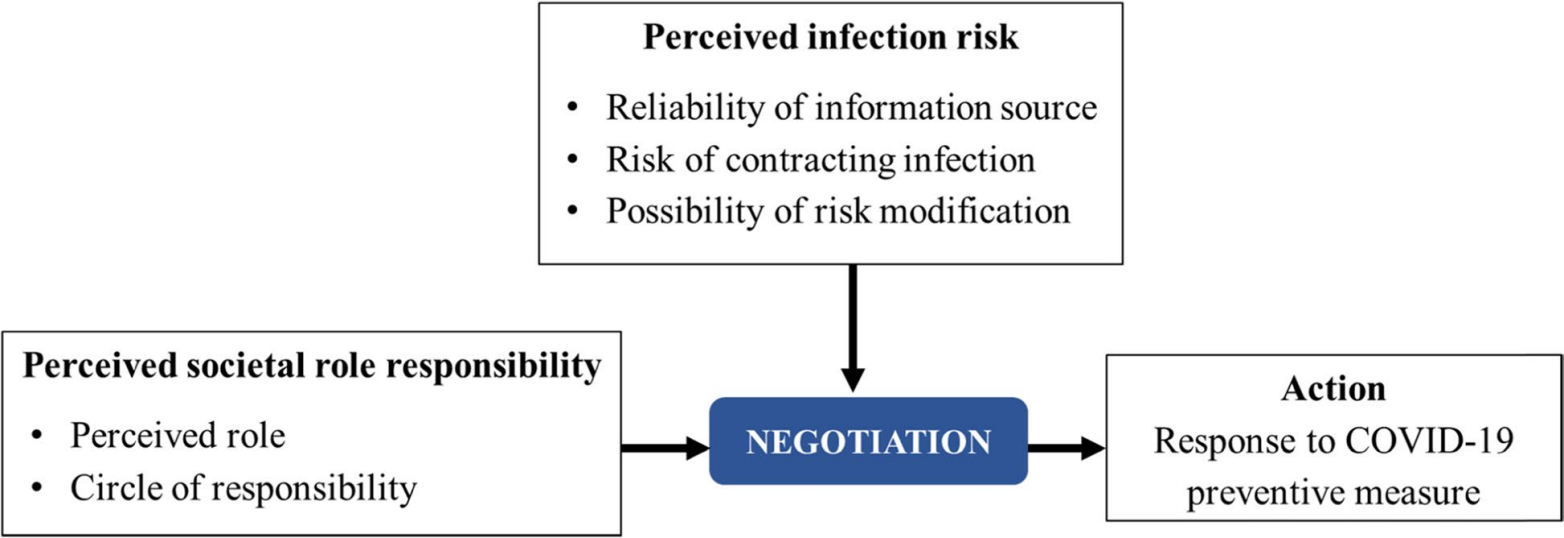
Public response to preventive measures from social responsibility perspective during the COVID-19 outbreak

Figure 2 shows the theoretical model, which illustrates that the individuals’ actions of social responsibility are a result of the intersection between perceived infection risk and societal role responsibility. These two are negotiated. The individuals’ perceived infection risk is constructed from their response to the information received and their self-efficacy in risk modification.

### Perceived societal role responsibility

The societal role is the perception of an individual. This role varies according to the context and current time since it depends on the type of relationship involved. A person can be a member of a workplace, family or society. Each role has a sense of responsibility attached to it, with obligations to be carried out by the individuals as members of the society. The type of relationship determines how far a person casts his or her circle of responsibility. There are two dimensions of societal role responsibility: the perceived role and the circle of responsibility.

#### Perceived role

The perceived role, which comes with a set of expected behaviours and conduct, influences a person’s decision in determining the best action [25]. A person can and often has multiple roles, but they only assume one role at a moment in time. For example, a person could be a mother who may also be a healthcare worker and an event host. Depending on her role in each situation, she would assume the role of a mother or a healthcare worker and think of the best interest for her children in some situations, and patients in other situations respectively.

A groom’s mother who was expected to take care of the guests’ safety in a wedding function stated the following:

*“I am a mother, who was just about to organise my son’s wedding at that time. It was…not MCO yet, but it’s still in warning status…where we can still organise feast…. we’d like to hold the wedding…but in a safe way, as you would have to invite a lot of guests.”* (P09, female, 52 y/o).

If a person became the host for a social event, he or she would be responsible for the comfort and safety of the guests. Despite his or her desire to host the event during the outbreak, the person was driven to consider the safety of others. Therefore, on the basis of the guests’ safety, he or she might cancel the event.

A social event host who was expected to take responsibility for his guests’ safety expressed:

*“I wanted to keep it going but I was persuaded by my family to cancel it. I wasn’t really concerned about the epidemic, but it was getting serious and I got scared. What if the virus infected other people?”* (P05, male, 29 y/o).

Similarly, a grandmother perceiving her responsibility towards the health of her grandsons stated:

*“We always wear the mask, even for small kids, my little grandson. I wear it for him even though he doesn’t want to.”* (P23, female, 61 y/o).

#### Circle of responsibility

The circle of responsibility depends on the size of the person’s definition of his or her social responsibility. There is a continuum of self, which is the smallest circle of responsibility, to as large a definition of society as one can perceive. The large circle of responsibility can stretch to include the global society and produce a more ‘collective effort’. The word ‘circle’ indicates that the circle can expand to a degree at the personal, society, national or global levels. Thus, the definition of this circle is personal, and defining it involves identifying the weight an individual would place on the importance of his or her personal goals such as health, enjoyment, life value, financial matters and societal relationship.

*“[I wear] a mask because we [healthcare provider] are contacting patient in hospital, so we have… to protect ourselves”* (P13, female, 33 y/o).

With the same act of adhering to public health measures, a large circle of responsibility expands beyond the self with the intention of avoiding spreading the disease to others.

*“When I’m back from work or the supermarket, I must clean myself before meeting my family. I’m staying with my mom. When I’m back, I’ll shower before meeting her because as an elder, she’s at-risk group.”* (P21, female, 45 y/o).

The “perceived societal role responsibility” cannot, by itself, explain a person’s societal behaviour regarding the pandemic; however, it forms the foundation for negotiation with a person’s perceived infection risk for an action he or she would take for preventive measures. While an intention to act may be present, the decision to act is balanced with the perceived infection risk.

### Perceived COVID-19 infection risk

Since the content of pandemic information includes risk of exposure and the protective measures, the perceived infection risk among members of the society is an outcome of an individual’s assessment of how high the risk of contracting the infection and the possibility of reducing the risk through protective measures. Before the government announced COVID-19 mitigation strategies, some individuals developed early risk perception and practised preventive measures after discovering local disease spread. However, other individuals perceived the risk only when the government enforced an MCO, indicating an imminent risk of infection. With increasingly government intervention, which indicated a widespread disease transmission, individuals began to perceive a higher risk of infection and considered reducing the risk through some protective measures.

*“When it began to spread here [in Malaysia] at that time…two weeks before the Prime Minister announced [the MCO], I was already scared of…this virus. Then, I started wearing a mask.”* (P07, female, 61 y/o).

*“I was a person that not really taking serious about this [pandemic], but when the government started to announce the lock-down or the movement restriction, that kind of [action] make me nervous… [that was] the point where I think… I have changed from not care to… give more concerned about it.”* (P05, male, 29 y/o).

How an individual comprehends the disease risk depends on a few factors:

#### Reliability of information source

Insight into COVID-19 serves as the starting point from which an individual understands the risk and determines how to respond. Initially, COVID-19 information was available from multiple sources, allowing a person to create his or her personal perception of the information. The source and type of COVID-19 information varied, each carrying a different weight and reliability as perceived by the individual. At one end of the information spectrum is the verified information from credible sources such as announcements by health authorities and global news. An individual tends to value such information, especially the information received from recognised sources and media.

*“I used to buy newspapers every day. So, usually I read papers, and then you see in the TV, the CNN and…other news.”* (P02, male, 73 y/o).

In contrast, unverified social media such as Twitter, WhatsApp and Facebook are also sources of information for the public. However, the information from these sources carried less weight and was perceived as being less reliable.

*“At that time, they (from Facebook) said that [MAEPS*^*8,24*^*] was for those who came back from overseas (quarantine centre). Then, I WhatsApp my sister…because those who stayed in Malaysia knew better about it (to verify the information).”* (P23, female, 61 y/o).

The source and type of COVID-19 information ranged from newspaper reports to individual opinions from respectable professionals. These sources were considered in variable weight and reliability.

*“We have a number of people in our group…who are politicians and doctors. There are public health doctors in the group as well. All of us… [thought that] it’s okay to go to Australia… after all, Australia is safer than Malaysia. All the reports [showed that]… all the cases in Australia were in Sydney, Gold Coast, not in Perth. We were prepared to go actually…”* (P03, female, 64 y/o).

#### The perceived risk of contracting the infection from public exposure

Risk through public exposure is perceived as how easy an individual could contract the infection while in public places. An individual perceived risk based on risk characteristics such as the infectious nature of the disease, proximity of infection based on the geographical spread, the similarity of social-cultural factors among infected individuals and one’s own susceptibility due to health status. A high perceived infection risk indicates that the infectious agent was easily spread, as well as positive cases were occurring in close proximity and in similar socio-cultural setting. A person also perceived high risk after assessing a location with high incidence of cases within their surroundings.

*“At first, when I looked at the Wuhan [condition]…I wasn’t afraid yet, I thought it only happened in Wuhan and wouldn’t reach Malaysia, it’s just Wuhan.”* (P07, female, 61 y/o).

*“Once it reached Malaysia, I mean, Wuhan and Malaysia are far from each other. So, when it reached Malaysia, it’s quite bad… I assumed that it could be prolonged and turn into a pandemic.”* (P11, female, 29 y/o).

*“I did not take this [COVID-19] seriously…and then there was actually…a new virus. They firstly say it comes out from the wild animals where they [people in China] eat wild animal… Malaysian less likely to take these wild animals.”* (P04, female, 33 y/o).

#### Possibility of risk modification through protective measures

An individual’s perceived infection risk could also be altered by their confidence in adopting some protection measures. The possibility of an individual engaging in risk modification is a personal evaluation of the effectiveness of protective measures, which may or may not be according to recommended guides or SOP. Confidence in protective measures is developed when the outcome of practising protective measures was evaluated and perceived as successful compared to the absence of protective measures.

*“I have confidence in preventive measures because…before this, my child went to a childcare centre…mingled with other kids, who had fever and everything. So, my child always gets a fever. When he was staying at home, he never had any fever. I believe in… the importance of social distancing to protect us from COVID-19.”* (P21, female, 45 y/o).

### The negotiation

Public response to the COVID-19 pandemic, which was reflected by their actions during the pandemic, was preceded by their corresponding intentions to act. The intentions were constructed from their perceived societal role responsibility. These intentions to finally complete the act were negotiated after they considered their perception of the risk of infection. As part of the ‘negotiation’, placing a heavy weight on one’s self in the societal role responsibility may tilt the balance towards an act that benefits one’s own agenda over infection risk. For instance, a person could undervalue infection risk over an important social event.

*“…but then again at that time, [it was] not so serious that you would protest against an assignment or project… So, that’s why I took the flight anyway.”* (P14, male, 33 y/o).

A person has the autonomy to decide what is best for himself or herself amidst the outbreak and the government’s restriction order. Hence, his or her decision also illustrated how far a person could align himself to societal goals without disregarding personal goals.

*“I’m worried because… the reception cannot be carried out. If we postpone [the wedding ceremony], we would never know when coronavirus is going to end. It could take one year, two years…five or six months. So, it would have been postponed due to an endless thing.”* (P18, female, 33 y/o).

**Fig. 3:**
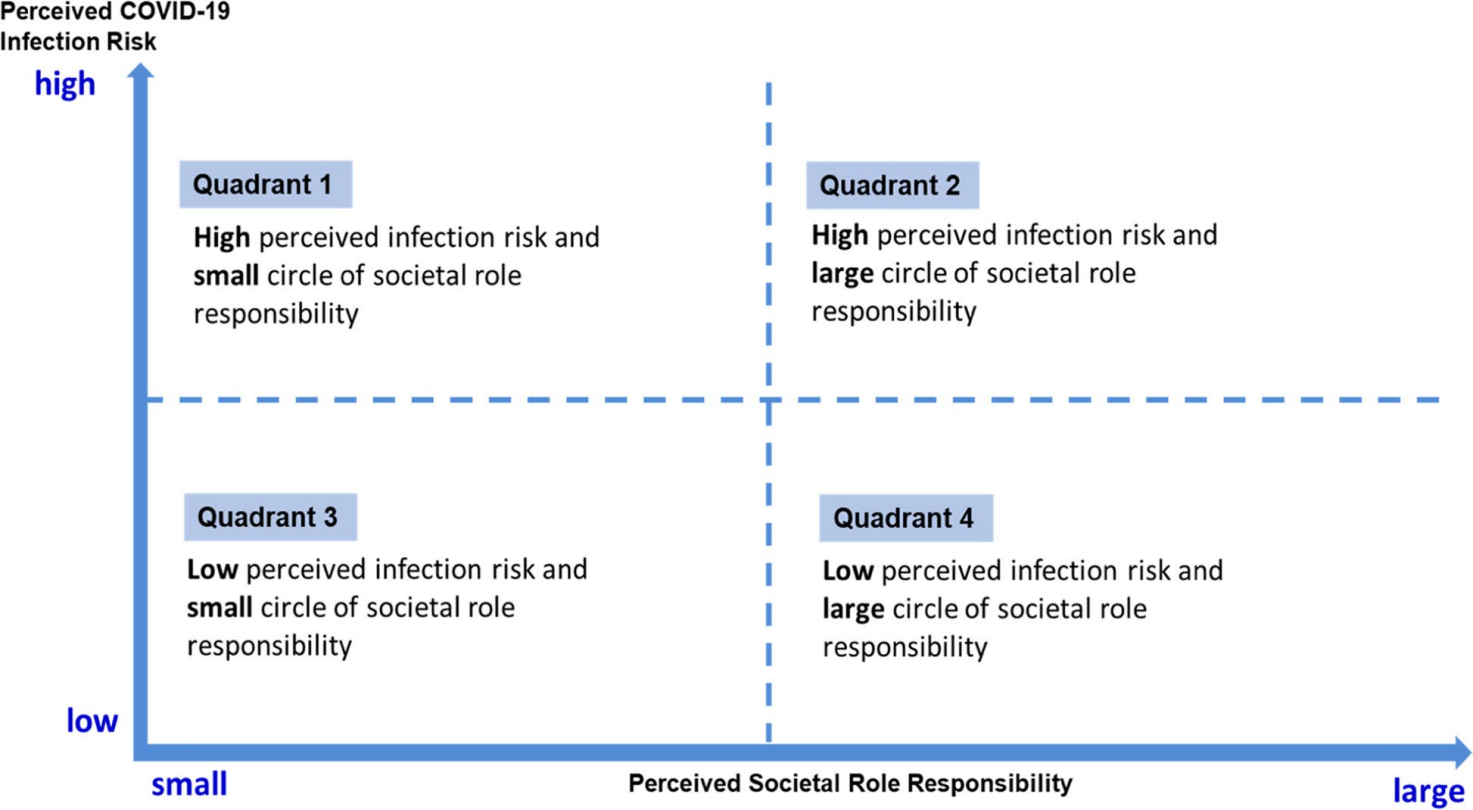
Intersection between perceived societal role responsibility and COVID-19 infection risk

In a different context, a person’s perceived societal roles vary, as does the perceived infection risk. Figure 3 illustrates these negotiations and the resulting range of actions from a spectrum quadrant of the the two factors of perceived societal roles and perceived infection risk. The spectrums include the following: perceived high infection risk and small circle of societal role responsibility; perceived high infection risk and large circle of societal role responsibility; perceived low infection risk and small societal role responsibility; and perceived low infection risk and large societal role responsibility.

#### Quadrant 1 Perceived high infection risk and perceived small circle of societal role responsibility

Perceived high risk of infection during a pandemic, places an individual at a “threatened” state of mind, thus invoking one’s “safeguarding” or “protecting” nature. However, from the perspective of a small role and circle of responsibility, the action was negotiated within the context of oneself and his or her safety, resulting in containing the action without intending to influence the behaviour of others.

*“We have to get prepared [to wear a mask]… because the awareness of people around us is not very high, so we have to protect ourselves.”* (P13, female, 33 y/o, perceived role: self).

#### Quadrant 2 Perceived high infection risk and perceived large societal role responsibility

Similarly, for a person who perceived a high infection risk with the perspective of a large role and circle of responsibility, the focus of his or her concerns expanded from oneself to others, especially those within the circle of care. The individual’s action was negotiated towards a proactive approach, aiming to mitigate others’ exposure to infection risk or influence others to reduce risky behaviour.

*“If I go [abroad] and know that Spain is already very badly affected, I don’t want to be one of the culprits that bring back the virus. I think that [is] very irresponsible… Even though I self-quarantine at home, I don’t think it’s good…”* (P06, female, 58 y/o, perceived role: a responsible citizen).

#### Quadrant 3 Perceived low infection risk and perceived small societal role responsibility

Perceived low risk of infection and small societal role responsibility placed an individual at ease and did not invoke the need to protect oneself or the society from the disease. As such, a perceived low infection risk of infection did not trigger an individual to adhere to preventive measures. The actions were negotiated for the benefit of oneself, guided by personal needs and interests. He or she was less concerned with the consequences of their actions on the society.

*“Well, there’s nothing to be afraid of… I had this sea license by [the authority] when [the police] asked me where to go; I would just tell him that I am going to catch cockle. Our hobby is to go out to sea…and no one spreads (virus) to you.”* (P17, male, 63 y/o, perceived role: self).

#### Quadrant 4 Perceived low infection risk and perceived large societal role responsibility

A person who perceived a low infection risk might not discern the necessity to negotiate a change of action or decision that has been made thus far. However, adequate information necessary for carrying out risk modification would be negotiated with the perceived large society role responsibility of a person. Eventually, some protective measures were adopted, and the benefit and safety of others would be taken into consideration.

*“…rumours said that MCO might be enforced. People began asking me if the wedding would be continued. Since there’s no announcement yet, if Allah wills, we would continue it but after the announcement, we updated them that we could only do the marriage ceremony specifically for closed family members.”* (P18, female, 33 y/o, perceived role: a host).

### Action – response to COVID-19 preventive measure

Action is the outcome of the negotiation between perceived societal role responsibility and perceived infection risk. An individual might take preventive measures by complying with the SOP such as wearing a mask, cancelling his or her social event, avoiding crowded places and reducing activities or social events. However, the notion of social responsibility for each action was shaped by the negotiation. As the conceptualisation of social responsibility differs across individuals, a person’s action during a pandemic may also vary contextually.

Another action a person can take is sharing information. In a specific situation, such as the COVID-19 outbreak, delivering information about the outbreak or preventive measures was similar to risk communication. However, there was a sense of control over information sharing. For example, a healthcare worker acted as a gatekeeper for information that he or she received directly from his or her work organisation. Whether or not the worker should share information with family members or friends depended on his or her personal decision.

*“Sometimes when I get news from MKN [National Security Council] or CPRC [National Crisis Preparedness and Response Centre] which I feel should be shared with my family members or friends, I will do that.”* (P08, female, 56 y/o).

A person’s action can also help prevent the spread of infection for self-protection or for public good. A person who perceived a larger societal role responsibility included the safety of oneself and others in his or her actions to prevent the spread of infection by adhering to specific SOPs.

*“The best thing is just to try to avoid gathering, because I got…two risk [groups]… My father-in-law…they are old….and my kids. So, I don’t want to be infected by this thing and bring it to my family.”* (P15, male, 35 y/o).

The individual’s action is generally similar for those who perceive high infection risk, but there is a variation in explaining their actions from the perspective of social role responsibility. He or she would generally adhere to guidelines during a pandemic. However, one’s actions vary considerably when he or she perceives low infection risk.

## Discussion

This study developed a substantive theoretical model to illustrate the process of how individuals responded to an early COVID-19 outbreak from the perspective of social responsibility. Individual response to COVID-19 was directly constructed from their perceived societal role responsibilities and further negotiated after considering the risk of infection. “Negotiation” was identified as an important intersection within this process. The different spectrums of social responsibility within an individual and among the society during the pandemic were noted to be the foundation for this negotiation to take place.

A pandemic urgently requires multi-disciplinary teams to work together, including and not limited to public health, clinical scientists, pharmaceutical industry and health policy specialists. The public also plays a significant role in mitigating the situation. Hence, understanding human behaviour is equally important since personal behaviour is a key factor in reducing the transmission of respiratory viruses [22]. Social scientists have acknowledged the importance of understanding the COVID-19 pandemic response from the social and behavioural lens and have highlighted some insights for outbreak preparedness [26]. Other public health frameworks such as the Health Belief Model [16, 21] and the COM-B Model [22] may provide behaviour diagnosis and reinforce mitigation behaviours during the outbreak of COVID-19 through careful arrangement of compliance with public health measures. The Health Belief model [21] focuses on the perceived threat, benefits, barriers and efficacy in mitigating the COVID-19 infection and focuses on the person’s perception of the disease; however, the health belief model does not incorporate society perspective although the pandemic is a condition closely related to the society [16]. The COM-B model has described a wide range of principles that can encourage individuals to engage in COVID-19 preventive behaviour [22]. The COM-B model indicates that preventive behaviour can only be practised when an individual has both the capability and the opportunity to show this behaviour; individuals are more motivated to choose personal protective behaviour. It shows that three components (Capability, Opportunity and Motivation) are closely linked to a person’s behaviour. COM-B model states that capability and opportunity are the primary parts of the model; however, during the MCO period, capability and opportunity were present, variation in behaviour remain.

Our model provides the additional contextual perspectives of the effect of infection risk, societal role and responsibilities perceived by individuals in negotiating behaviour for preventing COVID-19 infections. The government and many other authorised agencies may play a crucial role in influencing individual health behaviour but only to some extent. Our model implies that individuals exercise autonomy in determining which action to take when mitigating COVID-19 infection. Motivation to modify behaviour is contextual since it is derived from perceived societal role responsibility and infection risk. Our model provides a contextual relevance of undesirable behaviours. The MCO is seen as an undesirable event, and there was a tension between self and society as highlighted in our model. The important process of “negotiation” results in variation of behaviour, corresponding to perceived infection risk, whether low or otherwise. Importantly, our model presents a novel context – the context of pandemic; furthermore, it shows that undesirable behaviour is easy to engage with during the pandemic. Thus, capability and opportunity as highlighted in the COM-B model can be applicable during this situation. However, motivation varies across individuals, which is where our model offers substantive value.

Social responsibility is a perceived value which is significantly apparent across layers of society. However, application of social responsibility is commonly understood at the corporate level, which is defined as “any ‘responsible’ activity that allows a firm to achieve a sustainable competitive advantage, regardless of motive” [27, 28]. The individual perspective is equally important. From the same perspective, collective efforts in facilitating health improvement have gradually emphasised personal control over individual health behaviour through health education in order to create a collective social responsibility [29]. Such efforts were driven by the assumption that each individual could change societal norms through their habits or modifications of their lifestyles [30]. In 1986, the WHO promoted health as “a process of enabling people to increase control over, and to improve their health” [31]. The conventional health education is disease-oriented and has been used extensively in managing communicable and non-communicable diseases. In this study, we found the individual’s perspective was useful in analysing public health interventions during a pandemic situation. We found that an individual’s action is shaped by the tension between individual agenda and social responsibility, before the negotiation with perceived infection risk. This model provides a framework for Malaysian social responsibility during the COVID-19 outbreak and insights into understanding the interplaying elements. The time frame of the interviews was in the early stages of the outbreak in Malaysia, around the same time the MCO commenced; this was considered in the analysis of the findings. Nevertheless, these perceptions depict an early visualisation of individuals’ response to a pandemic and potentially shifts accordingly as the pandemic progresses.

### Policy implication, strengths and limitations

In terms of policy implication, our model explains that the action of a person during the early COVID-19 pandemic was the outcome of the “negotiation” between perceived societal role responsibility and perceived risk infection. In order to influence an individual’s response to an outbreak, the information provided to the public needs to articulate the exact role a person should play. Associations and organisations can empower members of the society by emphasising their potential roles during the pandemic and recommend actions. However, the spectrum of social responsibility in a different context indicates that human behaviour is complex, and a person’s action is influenced by the negotiation within a person. Hence, risk communication strategies could incorporate the element of negotiation, with clear SOPs that portray the importance of social responsibility during a pandemic. Authority figures could optimise various platforms to play their specific roles, such as an educator, adviser, or informer to alert society members and protect the public. Our model is useful for developing new norms during a pandemic and future intervention for behaviour change by emphasizing human negotiation behaviour.

By adopting a grounded theory approach, this study provides a substantive theory derived from emerging empirical data that attempts to explain the societal role responsibility during the COVID-19 outbreak. To enhance the theory, the sampling strategy used purposeful and theoretical sampling to ensure a maximum variation of demography and data collection within the context of an outbreak. In addition, the findings from this study can provide insight to various stakeholders, from health managers to policy makers, to strengthen preparedness for future outbreaks by understanding the spectrum of individual behaviours. The results from the ground up yielded valuable information with relevance to an outbreak context. Nevertheless, the context was in Malaysian, in the early stages of the outbreak, when resources and economic factors were not a major issue.

## Conclusions

The role of social responsibility has not been explored extensively in the academic literature, though it has been mentioned by country leaders and in risk communication materials. Our study adopted the grounded theory approach to develop a theoretical model that illustrates how individual response to early stage of COVID-19 preventive measure is the outcome of the negotiation between perceived societal role responsibility and perceived infection risk. The matrix diagram with the four types of behaviour facilitates the understanding of the abstract concept of negotiation in individual’s decision-making process. It also provides the spectrum of different types of behaviour in relation to public response to the COVID-19 pandemic. Although the model was conceptualised during the early stage of COVID-19 outbreak in Malaysia, we believe the model has relevance for adoption within the similar context of a disease outbreak.

## Data Availability

The dataset that support the findings of this article belongs to this study (The role of social responsibility during COVID-19 outbreak, NMRR-20-574-54389). At present, the data are not publicly available but can be obtained from the corresponding author and Head of Centre for Biostatistics & Data Repository, National Institutes of Health, Ministry of Health Malaysia on reasonable request and with the permission from the Director General of Health, Malaysia.

## Abbreviations

COM-B: Capability, Opportunity and Motivation Behaviour
IDI: in-depth interview
MCO: Movement Control Order
MERS-Cov: Middle East Respiratory Syndrome Coronavirus
SARS-CoV-2: Severe Acute Respiratory Syndrome Coronavirus 2
SOP: Standard Operating Procedure
WHO: World Health Organisation
